# Mucinous Histology Might Be an Indicator for Enhanced Survival Benefit of Chemotherapy in Stage II Colon Cancer

**DOI:** 10.3389/fmed.2020.00205

**Published:** 2020-06-05

**Authors:** Yong Huang, Kuanxue Ge, Guangshun Fu, Junfeng Chu, Wei Wei

**Affiliations:** ^1^Department of General Surgery, Jiangdu People's Hospital Affiliated to Medical College of Yangzhou University, Yangzhou, China; ^2^Department of Gastroenterology, Jiangdu People's Hospital Affiliated to Medical College of Yangzhou University, Yangzhou, China; ^3^Department of Radiotherapy, Jiangdu People's Hospital Affiliated to Medical College of Yangzhou University, Yangzhou, China

**Keywords:** mucinous histology, survival benefit, chemotherapy, stage II, colon cancer

## Abstract

**Background:** It was a difficult question to identify candidates who would benefit most from adjuvant chemotherapy in stage II colon cancer because of the paucity of relevant conclusive clinical trial results. We aimed to assess if mucinous adenocarcinoma (MUA) could be an indicator for the efficacy of adjuvant chemotherapy in stage II colon cancer.

**Methods:**Using SEER^*^Stat software V.8.3.5, eligible patients were then recruited from the SEER database. A χ^2^ test was applied to compare the distribution of different categorical variables between nonmucinous adenocarcinoma (NMUA) and MUA groups. We then used the Kaplan–Meier method to analyze overall survival (OS) of different histological types in stage II colon cancer, and the log-rank test was then used to assess the OS differences. The Cox proportional regression risk models were also built in our analyses to eliminate potential crossed bias from other prognostic factors.

**Results:**A total of 50,065 patients diagnosed with stage II colon cancer were recruited from the SEER database from 2004 to 2011; all the patients were divided into two groups, including NMUA (*n* = 44,785) and MUA (*n* = 5,280). The Cox analysis of the histological type indicated that the survival difference between MUA and NMUA failed to reach statistical significance in stage II colon cancer (*P* = 0.360). In NMUA, patients treated with adjuvant chemotherapy were independently associated with 37.2% decreased risk of overall mortality compared with those not [hazard ratio (HR) = 0.628, 95% confidence interval (CI) = 0.601-1.656, *P* < 0.001]; in MUA, the number increased to 41.5% (HR = 0.585, 95% CI = 0.515-0.665, *P* < 0.001).

**Conclusions:**Our study showed that the survival difference between MUA and NMUA failed to reach statistical significance in stage II colon cancer. More importantly, our study had provided the first evidence that chemotherapy would offer higher survival improvement in MUA compared with NMUA in stage II colon cancer; mucinous histology might be an indicator for enhanced survival benefit of chemotherapy in stage II colon cancer.

## Introduction

Colorectal cancer is the most prevalent malignancy of gastrointestinal cancer (1). Currently, surgical resection is the only curative treatment in patients with colorectal cancer. After the curative resection, however, patients with colorectal cancer would still have a high rate of recurrence, which was up to 30–40% (2). Stage II colon, defined as T3-T4 without lymph node or distant metastases according to the eighth edition of the American Joint Committee on Cancer (AJCC) Tumor Node Metastasis (TNM) staging system, was reported to make up ~36% of all the colon cancer (3). However, it was reported that the recurrence rate of stage II disease was ~25%, and there had been a continuous debate about the efficacy of adjuvant chemotherapy in stage II colon cancer (4).

The paucity of relevant conclusive clinical trial results made it a challenging problem to identify those who would benefit most from adjuvant chemotherapy in stage II colon cancer (5–8). Despite this, the European Society for Medical Oncology had clinical recommendations of adjuvant chemotherapy for high-risk stage II colon cancer (with risk factors such as lymph nodes sampling <12, poorly differentiated tumor, vascular or lymphatic or perineural invasion, tumor presentation with obstruction or tumor perforation and pT4 stage) (9). And some previous studies also had the similar recommendations (5, 10).

Mucinous adenocarcinoma (MUA), composed of more than 50% extracellular mucins, was a histologic subtype of colorectal cancer (11), and it accounted for approximately 10% of colorectal cancer (12, 13). Mucinous adenocarcinoma had distinct clinicopathological features compared with nonmucinous adenocarcinoma (NMUA) (14), and it remained debatable whether MUA was an adverse prognostic factor in patients with colon cancer (15–17). Few prior studies listed MUA as a high-risk factor for the treatment of chemotherapy. We therefore conducted this large population-based study to assess if MUA could be an indicator for the efficacy of adjuvant chemotherapy in stage II colon cancer.

## Patient Population and Selection

Sponsored by the National Cancer Institute, the Surveillance, Epidemiology, and End Results (SEER) program is an authoritative population-based cancer surveillance program. It was established in 1973 and covered approximately 30% of the total US population in SEER-participating regions. The SEER program collected registry and patient information, including general demographics, disease stage, cancer incidence, surgical variables, pathological type, and survival data (1). The latest follow-up information of patients ended in 2016.

**Figure 1:**
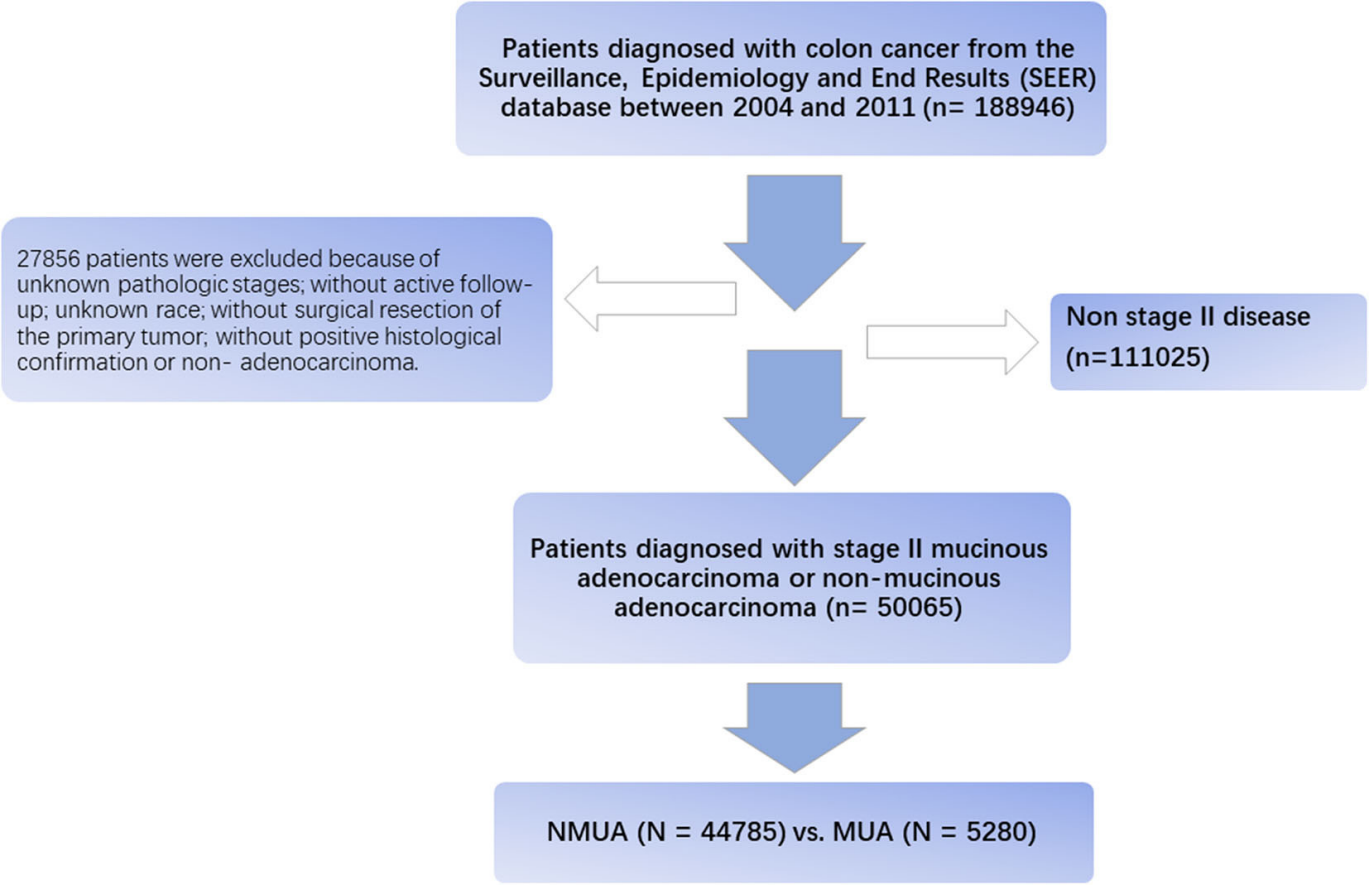
Flowchart of patients' cohort definition.

Using SEER^*^Stat software V.8.3.5, eligible patients were then recruited from the SEER database. The flow diagram of the data selection process is shown in Figure 1. First, patients diagnosed with colon cancer were selected from the SEER database between 2004 and 2011 (*n* = 188,946). We chose to select cases diagnosed before 2011 in order to obtain longer follow-up data. These patients were restaged according to the eighth edition of the AJCC TNM staging system, and node-negative patients with tumor deposit were redefined as N1. Then, 27,856 patients were excluded because of unknown pathologic stages, without active follow-up, unknown race, without surgical resection of the primary tumor, without positive histological confirmation, or nonadenocarcinoma. We also excluded patients with distant metastases or lymph node positivity; only patients diagnosed with stage II colon cancer were included in our analyses.

The following demographic and clinical characteristics were extracted from the SEER database:

T stage (T3 and T4), age at diagnosis (years), race (white, black, or other), gender (male and female), tumor site (cecum, ascending colon, hepatic flexure, transverse colon, splenic flexure, descending colon, and sigmoid colon), tumor grade (grade I/II, grade III/IV, or unknown), the receipt of chemotherapy (no/unknown or yes), and the histological type (NMUA or MUA).

### Statistical Analyses

All the eligible patients were divided into two groups, including NMUA and MUA groups. A χ^2^ test was applied to compare the distribution of different categorical variables between NMUA and MUA groups. The outcome of interest used in the current study was overall survival (OS). We then used the Kaplan–Meier method to analyze the OS according to the histology of colon cancer and the log-rank test was then used to assess the OS differences. In order to eliminate potential crossed bias from other prognostic factors, the Cox proportional regression risk models were also built in our analyses. Only those factors with *P* < 0.20 in univariate analyses would be included in the multivariate analyses. All the hazard ratios (HRs) of patient characteristics are shown with 95% confidence intervals (CIs). *P* < 0.05 was considered statistically significant. All statistical analyses were conducted using IBM SPSS, version 23 (IBM Corp., Armonk, NY, USA).

## Results

### Baseline Cohort Characteristics

A total of 50,065 patients diagnosed with stage II colon cancer were recruited from the SEER database from 2004 to 2011. All the patients were divided into two groups, including NMUA (*n* = 44,785) and MUA (*n* = 5,280). The median follow-up time of the whole cohort was up to 74 months, which was more than 5 years. Among the whole cohort, 24,173 patients (48.3%) were male and 25,889 patients (51.7%) were female. The median age was 73 years. And the pathological tumor stage showed that 43,533 patients (87.0%) were in the T3 stage, and 6,532 patients (13.0%) were in the T4 stage. Of all the patients, 16.2% (*n* = 8,135) of them had been treated with chemotherapy.

The descriptive patient characteristics of eligible patients were compared between NMUA and MUA (Table 1). It was found that MUA was more likely to be correlated with T4 stage (*P* < 0.001), older age (*P* = 0.009), white race (*P* < 0.001), female (*P* < 0.003), proximal colon (*P* < 0.001), and the receipt of chemotherapy with a borderline *P* value (*P* = 0.048).

**Table 1:** Demographic and patient characteristics according to the histology in stage II colon cancer.

**Characteristics**	**Number of patients (%)**		***P***
	**NMUA (*n* = 44,785)**	**MUA (*n* = 5,280)**	
**T stage**			<0.001
T3	39,136 (87.4)	4,397 (83.3)	
T4	5,649 (12.6)	883 (16.7)	
**Age (years)**			0.009
≤ 65	14,297 (31.9)	1,592 (30.2)	
>65	30,488 (68.1)	3,688 (69.8)	
**Race**			<0.001
White	36,655 (81.1)	4,512 (85.5)	
Black	4,902 (10.9)	502 (9.5)	
Other	3,228 (7.2)	266 (5.0)	
**Gender**			0.003
Male	21,729 (48.5)	2,447 (46.3)	
Female	23,056 (51.5)	2,833 (53.7)	
**Tumor location**			<0.001
Cecum	10,260 (22.9)	1,559 (29.5)	
Ascending colon	9,995 (22.3)	1,439 (27.3)	
Hepatic flexure	2,818 (6.3)	371 (7.0)	
Transverse colon	5,281 (11.8)	693 (13.1)	
Splenic flexure	1,931 (4.3)	230 (4.4)	
Descending colon	2,987 (6.7)	284 (5.4)	
Sigmoid colon	11,513 (25.7)	704 (13.3)	
**Grade**			<0.001
Grade I/II	36,426 (81.3)	4,046 (77.2)	
Grade III/IV	7,746 (17.3)	879 (16.6)	
Unknown	613 (1.4)	325 (6.2)	
**Chemotherapy**			0.048
No/unknown	37,558 (83.9)	4,372 (82.8)	
Yes	7,227 (16.1)	908 (17.2)	

### Prognostic Value of MUA in Stage II Colon Cancer

**Figure 2:**
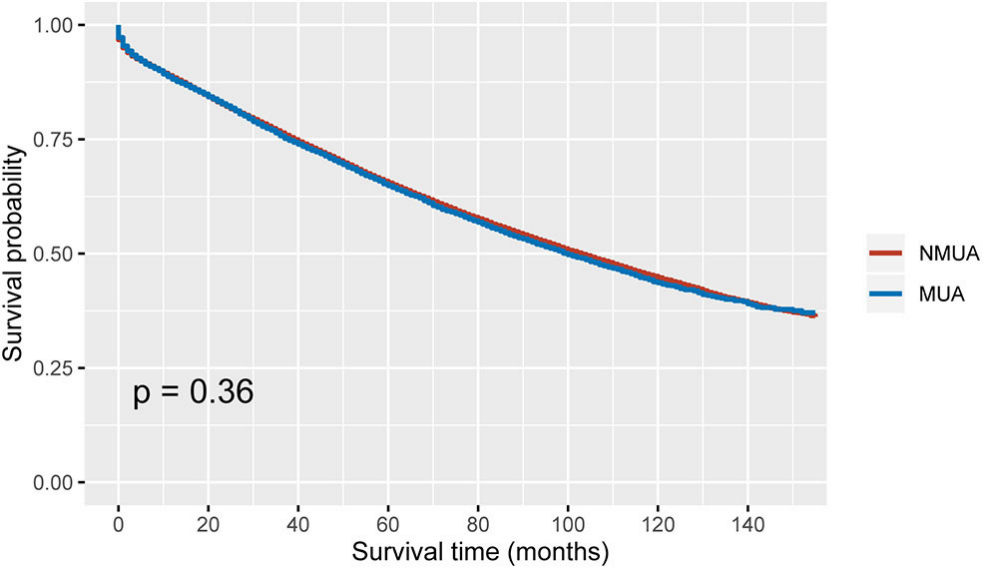
Kaplan–Meier curves for overall survival in stage II colon cancer.

As shown in Figure 2, stage II MUA had similar OS as compared with stage II NUMC (*P* = 0.360). The 5-year OS rates of MUA and NMUA were 64.9 and 65.4%, respectively. Table 2 shows the results of univariate analyses of characteristics including T stage, age at diagnosis, race, gender, tumor site, tumor grade, the receipt of chemotherapy, and the histological type. Those clinicopathological factors with *P* < 0.20 in univariate analyses were included in multivariate analyses: T stage (*P* < 0.001), age at diagnosis (*P* < 0.001), race (*P* < 0.001), gender (*P* = 0.001), tumor site (*P* < 0.001), tumor grade (*P* < 0.001), and the receipt of chemotherapy (*P* < 0.001). However, univariate Cox analysis of the histological type indicated that the survival difference between MUA and NMUA failed to reach statistical significance in stage II colon cancer (*P* = 0.360). And we can also see that patients treated with adjuvant chemotherapy were independently associated with 37.8% decreased risk of overall mortality compared with patients without the receipt of adjuvant chemotherapy in stage II colon cancer (HR = 0.622, 95% CI = 0.597-0.648, *P* < 0.001).

**Table 2:** Univariate and multivariate analyses of prognostic factors in stage II colon cancer.

**Variable**	**Univariate analyses**		**Multivariate analyses**	
	**HR (95% CI)**	***P***	**HR (95% CI)**	***P***
**Histology**		0.360		
NMUA				
MUA				
**T stage**		<0.001		<0.001
T3			1	
T4			1.692 (1.635-1.752)	
**Age (years)**		<0.001		<0.001
≤ 65			1	
>65			3.073 (2.968-3.182)	
**Race**		<0.001		<0.001
White			1	
Black			1.116 (1.071-1.162)	<0.001
Other			0.715 (0.676-0.756)	<0.001
**Gender**		0.001		<0.001
Male			1	
Female			0.874 (0.853-0.897)	
**Tumor location**		<0.001		<0.001
Cecum			1	
Ascending colon			0.984 (0.949-1.021)	0.392
Hepatic flexure			1.026 (0.972-1.084)	0.353
Transverse colon			1.100 (1.054-1.149)	<0.001
Splenic flexure			1.126 (1.057-1.201)	<0.001
Descending colon			1.067 (1.009-1.128)	0.023
Sigmoid colon			1.095 (1.056-1.135)	<0.001
**Grade**		<0.001		<0.001
Grade I/II			1	
Grade III/IV			1.124 (1.088-1.161)	<0.001
Unknown			1.185 (1.087-1.292)	<0.001
**Chemotherapy**		<0.001		<0.001
No/unknown			1	
Yes			0.622 (0.597-0.648)	

### Mucinous Histology Might Be an Indicator for Enhanced Survival Benefit of Chemotherapy in Stage II Colon Cancer

**Figure 3:**
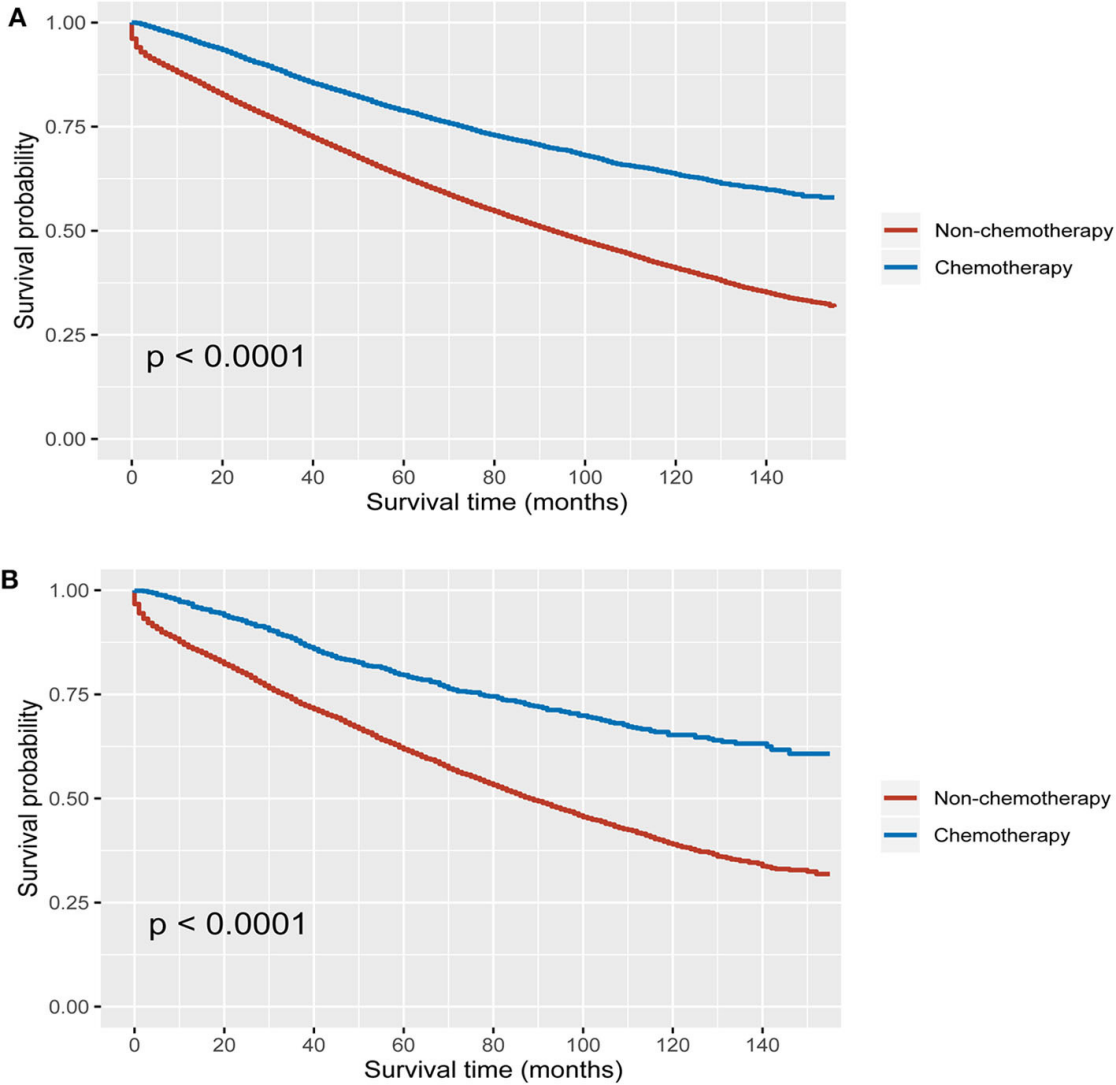
Kaplan–Meier curves for overall survival in stage II colon cancer ith **(A)** MUA, **(B)** NMUA.

**Figure 4:**
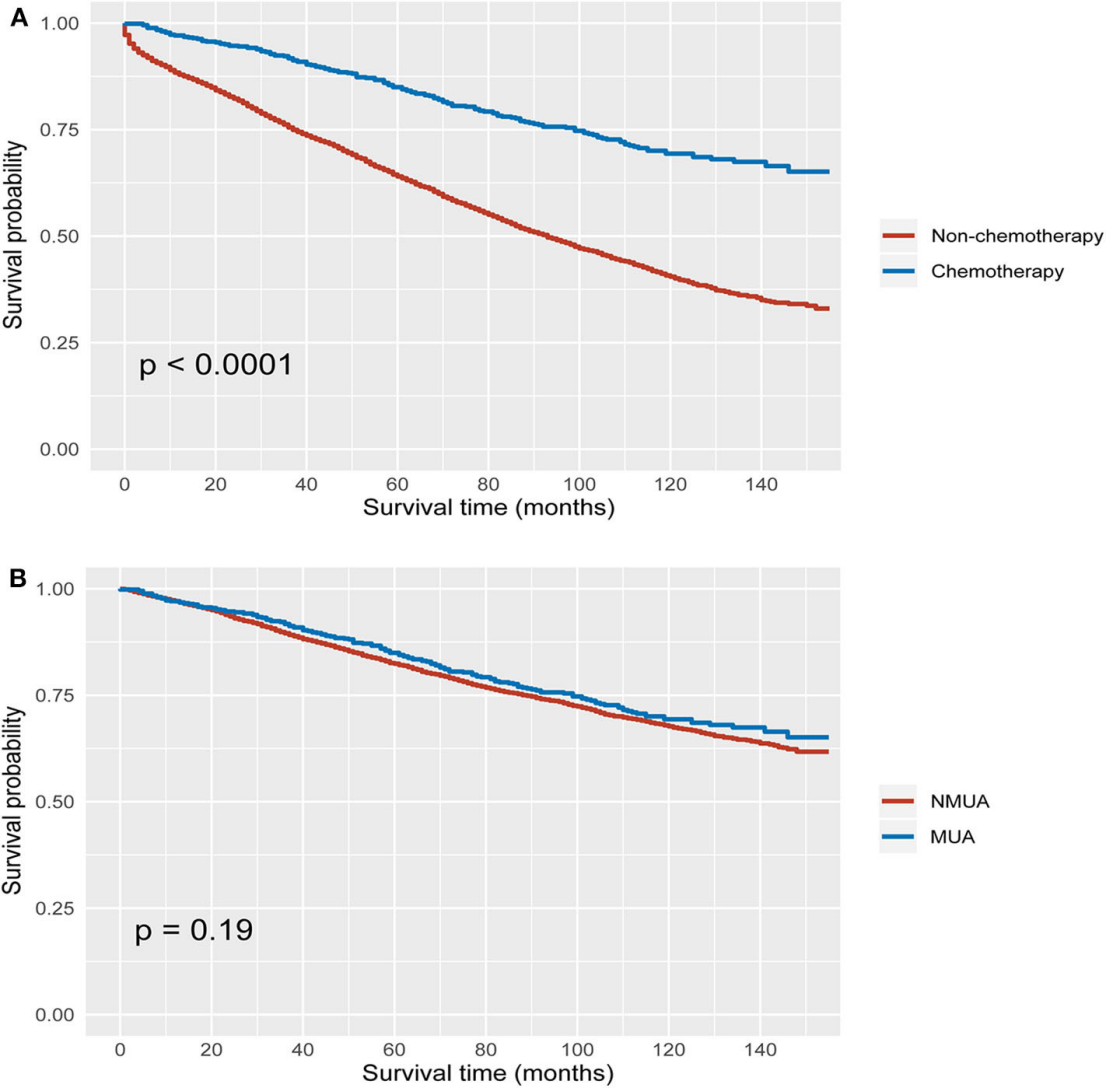
Kaplan–Meier curves for overall survival in stage II colon cancer with **(A)** pT3 N0 M0 MUA, **(B)** pT3 N0 M0 colon cancer with the receipt of chemotherapy.

Using Kaplan–Meier method, it was found that the receipt of chemotherapy offered significantly improved OS in both MUA and NMUA groups. In NMUA, the 5-year OS rates of chemotherapy and nonchemotherapy groups were 78.8 and 62.9%, respectively (*P* < 0.0001, Figure 3A). In MUA, the 5-year OS rates of chemotherapy and nonchemotherapy groups were 79.6 and 61.8%, respectively (*P* < 0.0001, Figure 3B). The aforementioned results indicated that the receipt of chemotherapy had 15.9% increased 5-year OS rate compared with nonchemotherapy in NMUA, whereas the efficacy of chemotherapy seemed to be more obvious in MUA: the receipt of chemotherapy had 17.8% increased 5-year OS rate compared with nonchemotherapy in MUA. In addition, we also conducted the subgroup analyses in pT3 N0 M0 colon cancer. It was found that the receipt of chemotherapy also offered significantly improved OS in pT3 N0 M0 MUA, and the 5-year OS rates of chemotherapy and nonchemotherapy groups were 92.0 and 89.6%, respectively (*P* < 0.0001, Figure 4A); however, the receipt of chemotherapy did not offer OS difference between pT3 N0 M0 MUA and pT3 N0 M0 NMUA (*P* = 0.19, Figure 4B).

Cox proportional hazards models were then employed to verify the above findings. Table 3 shows the results of univariate analyses of characteristics including T stage, age at diagnosis, race, gender, tumor site, tumor grade, the receipt of chemotherapy and the histological type in stage II NMUA, and those clinicopathological factors with *P* < 0.20 in univariate analyses were included in multivariate analyses: T stage (*P* < 0.001), age at diagnosis (*P* < 0.001), race (*P* < 0.001), gender (*P* < 0.001), tumor site (*P* < 0*P* < 0.001), tumor grade (*P* < 0.001), and the receipt of chemotherapy (*P* < 0.001). The results of multivariate analyses showed that patients treated with adjuvant chemotherapy were independently associated with 37.2% decreased risk of overall mortality compared with those not in stage II colon cancer (HR = 0.628, 95% CI = 0.601–1.656, *P* < 0.001).

**Table 3:** Univariate and multivariate analyses of prognostic factors in stage II NMUA.

**Variable**	**Univariate analyses**		**Multivariate analyses**	
	**HR (95% CI)**	***P***	**HR (95% CI)**	***P***
**T stage**		<0.001		<0.001
T3			1	
T4			1.715 (1.652-1.779)	
**Age (years)**		<0.001		<0.001
≤ 65			1	
>65			3.027 (2.918-3.140)	
**Race**		<0.001		<0.001
White			1	
Black			1.125 (1.078-1.174)	<0.001
Other			0.714 (0.674-0.757)	<0.001
**Gender**		<0.001		<0.001
Male			1	
Female			0.870 (0.847-0.893)	
**Tumor location**		<0.001		<0.001
Cecum			1	
Ascending colon			0.976 (0.939-1.014)	0.210
Hepatic flexure			1.024 (0.965-1.085)	0.435
Transverse colon			1.095 (1.046-1.147)	<0.001
Splenic flexure			1.108 (1.035-1.185)	0.003
Descending colon			1.046 (0.986-1.109)	0.133
Sigmoid colon			1.084 (1.044-1.127)	<0.001
**Grade**		<0.001		<0.001
Grade I/II			1	
Grade III/IV			1.133 (1.095-1.173)	<0.001
Unknown			1.203 (1.081-1.339)	0.001
**Chemotherapy**		<0.001		<0.001
No/unknown			1	
Yes			0.628 (0.601-1.656)	

Table 4 shows the results of univariate analyses of characteristics including T stage, age at diagnosis, race, gender, tumor site, tumor grade, the receipt of chemotherapy, and the histological type in stage II MUA, and those clinicopathological factors with *P* < 0.20 in univariate analyses were included in multivariate analyses: T stage (*P* < 0.001), age at diagnosis (*P* < 0.001), race (*P* < 0.001), tumor grade (*P* = 0.056), and the receipt of chemotherapy (*P* < 0.001). However, both gender (*P* = 0.261) and tumor site (*P* = 0.892) did not show enough prognostic value in univariate analyses. The results of multivariate analyses showed that patients treated with adjuvant chemotherapy were independently associated with 41.5% decreased risk of overall mortality compared with those not in stage II colon cancer (HR = 0.585, 95% CI = 0.515-0.665, *P* < 0.001).

**Table 4:** Univariate and multivariate analyses of prognostic factors in stage II MUA.

**Variable**	**Univariate analyses**		**Multivariate analyses**	
	**HR (95% CI)**	***P***	**HR (95% CI)**	***P***
**T stage**		<0.001		<0.001
T3			1	
T4			1.557 (1.410-1.718)	
**Age (years)**		<0.001		<0.001
≤ 65			1	
>65			3.415 (3.049-3.824)	
**Race**		<0.001		0.014
White			1	
Black			1.031 (0.899-1.183)	0.663
Other			0.738 (0.599-0.909)	0.004
**Gender**		0.261		
Male				
Female				
**Tumor location**		0.892		
Cecum				
Ascending colon				
Hepatic flexure				
Transverse colon				
Splenic flexure				
Descending colon				
Sigmoid colon				
**Grade**		0.056		0.222
Grade I/II			1	
Grade III/IV			1.030 (0.931-1.139)	0.572
Unknown			1.139 (0.980-1.324)	0.089
**Chemotherapy**		<0.001		<0.001
No/unknown			1	
Yes			0.585 (0.515-0.665)	

## Discussion

As a rare and special type of colorectal cancer, MUA was composed of more than 50% extracellular mucins and had distinct clinicopathological features compared with NMUA. It was reported that MUA was more likely to be associated with advanced stages in colorectal cancer and was less responsive to chemotherapy compared to NMUA (12, 18), but it remained debatable whether MUA was an adverse prognostic factor in patients with colon cancer (15–17). In stage II colon cancer, however, the finding that survival difference between MUA and NMUA was not statistically different was gradually becoming clear.

In 2011, a study involved 1,025 unselected patients from Italy showed that the OS of stage II colon cancer with MUA was not significantly different from those with NMUA (*P* = 0.206) (19). Later, in 2018, a study also found that the cancer-specific survival difference between MUA and NMUA was not statistically significant (*P* = 0.597) (20). Recently, Fields et al. (21) conducted a retrospective analysis and reported that the 5-year survival rates of patients with stage II NMUA and MUA were 65.1 and 63.5%, respectively, and the survival difference achieved statistical significance (*P* = 0.002). In adjusted analysis, however, they found there was no significant difference in survival between NMUA and MUA patients in stage II disease.

In our study, it was shown that MUA was more likely to be correlated with T4 stage (*P* < 0.001), which had been demonstrated in prior studies (12, 18). The Kaplan–Meier analyses showed that stage II MUA had similar OS as compared with stage II NMUA (*P* = 0.360), and the 5-year OS rates of MUA and NMUA were 64.9% and 65.4%, respectively. Moreover, the results of Cox analysis also indicated that the survival difference between MUA and NMUA failed to reach statistical significance in stage II colon cancer (*P* = 0.360). The 5-year OS rates of MUA and NMUA were similar to previous report (19). More importantly, we showed again no survival difference between MUA and NMUA in stage II colon cancer, even in pT3 N0 M0 colon cancer with receipt of chemotherapy

Despite clinical guidelines that had recommendations of adjuvant chemotherapy for high-risk colon cancer, the efficacy of adjuvant chemotherapy in high-risk disease had always been the subject of debate (22–24), indicating the necessity to identify candidates for adjuvant chemotherapy in stage II colon cancer. Few prior studies listed MUA as a high-risk factor. We therefore conducted this large population-based study to assess if MUA could be an indicator for the efficacy of adjuvant chemotherapy in stage II colon cancer. The results of our study indicated that the receipt of chemotherapy had 15.9% increased 5-year OS rate compared with nonchemotherapy in NMUA (the 5-year OS rates of chemotherapy and nonchemotherapy groups were 78.8 and 62.9%, respectively, *P* < 0.0001) However, the efficacy of chemotherapy seemed to be more obvious in MUA: the receipt of chemotherapy had 17.8% increased 5-year OS rate compared with nonchemotherapy in MUA (the 5-year OS rates of chemotherapy and nonchemotherapy groups were 79.6 and 61.8%, respectively, *P* < 0.0001). The results of multivariate Cox analyses showed that patients treated with adjuvant chemotherapy were independently associated with 37.2% decreased risk of overall mortality compared with patients without the receipt of chemotherapy in stage II NMUA. However, adjuvant chemotherapy had 41.5% independently decreased risk of overall mortality in stage II NMUA, showing that the therapeutic effect of chemotherapy had been enhanced in MUA.

To our knowledge, few previous studies focused on the response to chemotherapy in stage II MUA (20, 21). In a recent study, Fields et al. (21) performed a retrospective analysis to evaluate the survival benefit of chemotherapy in patients with stages II and III colon cancer with the histology of MUA. The researchers reported that there was a significant survival difference between patients undergoing chemotherapy and those not undergoing chemotherapy (HR = 0.79, 95% CI = 0.69–0.90, *P* < 0.001) in stage II MUA. Therefore, they concluded that adjuvant chemotherapy had significantly improved OS compared to those not undergoing chemotherapy in stage II colon cancer with the histology of MUA. In addition, Hu et al. (20) reported that the disease-free survival was identical in MUA and adenocarcinoma patients after neoadjuvant chemotherapy and believed the histology of MUA can be used as a high-risk factor in stage II colorectal cancer patients. The two researches supported the receipt of chemotherapy in stage II MUA; however, both of them did not have direct comparison of the chemosensitivity between stage II colon cancer with MUA and NMUA. And Hu et al. (20) had mixed colon cancer patients and rectal cancer patients together, which would impact the clinical application of study results in stage II colon cancer.

Recently, Rosati et al. (25) recruited 474 patients from 94 centers enrolled in the per-protocol population defined in the TOSCA trial. They found that the evaluation of MUA histology could be considered an indicator for longer chemotherapy treatment in stage II colon cancer, which was consistent with our study. The response of chemotherapy, however, was still debated in the histologic type of MUA. Some studies have shown that MUA had a poor response to chemotherapy, whereas others have shown a survival benefit of chemotherapy in MUA (26–29). In stage II colon cancer, therefore, our study had provided the first evidence that chemotherapy would offer higher survival improvement in MUA compared with NMUA in stage II colon cancer. Mucinous adenocarcinomas have higher chemotherapy sensitivity in stage II disease as compared with other tumor stages, and mucinous colon cancer treatment algorithms should take staging information into account.

Our study has two important limitations. First, though SEER database was a database with reliable information, inevitably, it has some inherent limitations with the lack of some features including the types and doses of chemotherapy and information on tumor relapse, and it did not pay enough attention to molecular biology of colon cancer. Second, our research was a retrospective one, which had inherent deficiencies that could lead to confusion or observer bias.

## Conclusions

Our study showed that the survival difference between MUA and NMUA failed to achieve statistical significance in stage II colon cancer. More importantly, our study had provided the first evidence that chemotherapy would offer higher survival improvement in MUA compared with NMUA in stage II colon cancer, indicating MUA had higher chemotherapy sensitivity in stage II disease as compared with other tumor stages, and mucinous colon cancer treatment algorithms should take staging information into account. Mucinous histology might be an indicator for enhanced survival benefit of chemotherapy in stage II colon cancer.

## Data Availability Statement

Publicly available datasets were analyzed in this study. This data can be found here: https://seer.cancer.gov/.

## Author Contributions

WW conceived and designed the study. YH and KG collected and analyzed the data. YH, KG, and GF performed the statistical analysis. GF and JC wrote the first draft of the manuscript. YH, KG, and WW revised the final data and the manuscript. All the authors reviewed the final draft of the manuscript and approved the submitted version.

## Conflict of Interest

The authors declare that the research was conducted in the absence of any commercial or financial relationships that could be construed as a potential conflict of interest.
